# Safety and effectiveness of nafamostat mesylate in continuous renal replacement therapy in patients with sepsis-associated acute kidney injury: a prospective randomized controlled trial

**DOI:** 10.1515/med-2025-1369

**Published:** 2026-02-24

**Authors:** Yu Chen, Fang Feng, Qun Li, Huyong Yang, Weiwei Yang

**Affiliations:** Department of Critical Care Medicine, Gansu Provincial Central Hospital, Lanzhou, Gansu, China; Department of Critical Care Medicine, The Second Hospital and Clinical Medical School, Lanzhou University, Lanzhou, Gansu, China; Department of Critical Care Medicine, People’s Hospital of Linxia State, Linxia, Gansu, China

**Keywords:** sepsis, acute kidney injury, continuous renal replacement therapy nafamostat mesylate, critical care medicine

## Abstract

**Objectives:**

To determine the safety and effectiveness of the anticoagulation regimen nafamostat mesylate for continuous renal replacement therapy in patients with sepsis-associated AKI.

**Methods:**

According to the inclusion and exclusion criteria, a total of 42 patients with sepsis and complicated renal function impairment who required CRRT were included. Among them, 2 patients were excluded because their admission time was less than 24 h. Finally, 40 patients were included. The Nafamostat mesylate anticoagulation method was selected for the experimental group and regional citrate anticoagulation was used in the control group.

**Results:**

At admission (experimental group vs. control group), the following findings were obtained: sCr: 391.2 ± 1.94 vs. 389.7 ± 1.46 ummol/L, BUN: 12.97 ± 1.51 vs. 11.86 ± 1.75 mmol/L, APTT: 23.43 ± 1.27 vs. 24.12 ± 1.53 s, and Plt: 80.15 ± 12.27 vs. 83.15 ± 10.35 × 109/L. Four hours after CRRT treatment, the following findings were obtained: sCr: 280.85 ± 1.89 vs. 283.41 ± 1.46 ummol/L, BUN: 12.02 ± 1.55 vs. 11.86 ± 1.75 mmol/L, APTT of prefilter: 41 ± 3.89 vs. 25.31 ± 2.17 s, postfilter: 81.63 ± 3.76 s, Plt: 80.2 ± 13.04 vs. 83.15 ± 10.35 × 109/L, and pH 7.37 ± 0.03 vs. 7.41 ± 0.02. A comparison of sCr and APTT (prefilter) revealed that p<0.05 was statistically significant, but there was no significant difference in the other indicators. In the control group, citrate accumulation occurred in 2 patients; for a total calcium/arterial calcium ion concentration >2.5, there were no obvious adverse reactions. The lifespans of the first filter were 60.2 ± 10.09 and 59.07 ± 11.44 h, and the ICU lengths were 7.35 ± 1.35 and 7.21 ± 1.17 days, Nafamostat mesylate prolonged filter lifespan compared to RCA, however, ICU LOS was same.

**Conclusions:**

The anticoagulation regimen of nafamostat mesylate may be safe and effective in patients with sepsis-associated AKI receiving CRRT.

## Background

Sepsis-associated acute kidney injury (sepsis-associated AKI) is a common life-threatening complication in hospitalized and critically ill patients. sepsis-associated AKI increases in-hospital mortality by 6–8 times [1] and increases the risk of progression to chronic kidney disease (CKD) by 3-fold [2], 3]. In addition, up to 1/4 of sepsis-associated AKI patients require renal replacement therapy (RRT) [4].

Continuous renal replacement therapy (CRRT) has become the treatment of choice for acute kidney injury, especially for critically ill patients with haemodynamic instability [5]. Anticoagulation is necessary for the effective delivery of CRRT, but this requirement can also pose challenges because many critically ill patients with sepsis already present with increased risks of bleeding and coagulation. However, if anticoagulant therapy is not administered during CRRT, the lifespan of the filter and tubing will be reduced, resulting in a reduced therapeutic effect and an increased cost of treatment [6].

Nafamostat mesylate is a synthetic serine protease inhibitor that can inhibit various enzymes in the coagulation system, fibrinolytic system, and complement system [7], [8], [9]. Nafamostat mesylate inhibits the activity of thrombin, factors VIIa, Xa, and XIIa and the anticoagulant effect is not influenced by the level and activity of antithrombin III. Two randomized controlled trials compared the anticoagulant effect and safety of nafamostat mesylate and no anticoagulant mode among high-risk bleeding patients, respectively, and found that nafamostat mesylate can significantly prolong the use of extracorporeal circulation tubes. However, there was no statistically significant difference in bleeding complications between the two groups [10], 11].

Ideal anticoagulation therapy should be easy to implement and monitor, have fewer adverse effects, and be cost effective. However, although there are currently guidelines for nafamostat mesylate for CRRT anticoagulation, the recommendation level is low, and there is no relevant consensus on its effective dose, monitoring indicators, or adverse reactions during CRRT anticoagulation. Currently, there are clinical studies on the use of nafamostat mesylate and unfractionated heparin for CRRT anticoagulation, but the results are inconsistent. A study conducted by Hwang SD [12] and others in 2017 showed that the lifespan of the filter in the nafamostat mesylate group was significantly longer than that in the heparin anticoagulation group (24.3 vs. 17.5 h, p<0.001), and APTT was prolonged. However, bleeding events did not increase. Also, the results of Makino S [13] showed that the nafamostat mesylate anticoagulation agent reduced the risk of bleeding (3.3 vs. 27.0 %, OR=0.09, p=0.04), but there was no difference in the lifespan of the filter compared with that in the unfractionated heparin anticoagulation agent group (25.5 vs. 30.5 h, p=0.16). Since nafamostadol mesylate is widely used in Japan and South Korea, there is currently a lack of clinical randomized controlled trials directly comparing nafamostadol mesylate with regional citrate anticoagulation agents, so its safety and effectiveness need to be further clarified. Therefore, this study aimed to apply nafamostat mesilate for anticoagulation during CRRT in patients with septic acute kidney injury. The safety and effectiveness of these agents should be further clarified.

## Materials and methods

### Clinical data

General clinical data were collected in tabular form for patients admitted to the hospital between May 2023 and September 2023 and who were diagnosed with sepsis complicated with acute kidney injury and who needed CRRT. The following data were collected: age, sex, acute physiology and chronic health II (APACHE II) score, site of infection, serum creatinine (sCr), blood urea nitrogen (BUN) levels at admission, activated partial thromboplastin time (APTT), platelet count (platelet, Plt), pH value, arterial calcium ion concentration (iCa), sCr, BUN, APTT (pre- and postfilter), calcium ion (pre- and postfilter), platelet count and pH value 4 h after CRRT.

### Design

A prospective, open-label, randomized controlled pilot trial was also conducted. Randomization was performed with the use of a centralized computer generated assignment sequence at the first day in the ICU. And block randomization was used as a method of random grouping. Complete randomization was applied in our study. First, patients were ranked according to the order of enrollment. A set of random numbers was then assigned to the patient in the same order. And then, rank the random number column from smallest to largest, with the first five for the experimental group and the last five for the control group. This trial was approved by the Ethics Committee of the Second Hospital and Clinical Medical School, Lanzhou University, and the ethics approval number is 2023A-454. Informed consent was signed by the patients or family members and was obtained from all included patients. (When the patient was unable to complete the signature due to the use of sedative drugs, coma, etc., his or her immediate family members signed the informed consent form.).

### Inclusion and exclusion criteria

The inclusion criterion was patients with sepsis who met the diagnostic criteria for sepsis 3.0, were complicated by acute kidney injury and required CRRT.

The exclusion criteria for patients were as follows: <18 years of age, pregnancy, acute or chronic liver failure or chronic kidney failure, DIC, COVID, anti-platelets used and an admission time <24 h.

### Methods

Patients diagnosed with sepsis complicated by acute renal injury who required CRRT were prospectively included from May 2023 to September 2023. All patients included in the study were given bundled treatment for sepsis (measurement of lactate levels, adequate fluid resuscitation, broad-spectrum antibiotics, vasoactive drugs (norepinephrine is preferred, when the dosage of norepinephrine is >20 μg/min, vasopressin 0.03 U/min is added) to maintain mean arterial pressure ≥65 mmHg). The treatment mode and parameters settings were as follows: a Fresenius Multifiltrate CRRT machine was used, the mode was continuous veno-venous haemodiafiltration (CVVHDF), the blood flow rate was 150∼180 mL/h, and the replacement fluid input method was postdilution. In both patients, the femoral vein (right side preferred) was used for indwelling double-lumen catheterization as the vascular access. Anticoagulation method: In the experimental group, nafamostat mesylate was continuous intravenous pumping (the preparation concentration of nafamostat mesylate was 1 mg/mL, the bolus dose was 10 mg, and the initial dose was 20 mg/h). The APPT value was maintained at 1.5–2 times the normal value, and the infusion amount was adjusted according to APTT. In the control group, the initial citrate dosage was 1.1 times the blood flow rate to maintain the postfilter free calcium ion level at 0.25–0.35 mmol/L and the arterial calcium ion concentration at 1–1.2 mmol/L. The infusion amount was adjusted according to the postfilter calcium ion concentration.

### Outcomes

①Lifespan of the first filter; ②incidence of new bleeding episodes; ③ICU length of stay.

### Ethics approval and consent to participate

This trial was approved by the Ethics Committee of the Second Hospital and Clinical Medical School, Lanzhou University, and the ethics approval number is 2023A-454. Informed consent was signed by the patients or family members and was obtained from all included patients. (When the patient was unable to complete the signature due to the use of sedative drugs, coma, etc., his or her immediate family members signed the informed consent form.).

### Consent for publication

Not applicable.

## Statistical analysis

SPSS 21.0 software (SPSS, Inc., Chicago, IL, USA) was used for the statistical analysis of the data. If the measured data displayed a normal distribution, the means ± standard deviations (*x* ± *s*) were reported. A t-test was used to compare the data between the two groups, and the q-q normal probability graph was used for the normality test. If the measured data did not display a normal distribution, the median (*M*) and interquartile ranges (QL, Qu) were used to present the measured data. Count data are reported as n (%). Pearson’s test was used to compare the dichotomous data between two groups, and the Mann‒Whitney U test was used for the ordered classification of multiple groups. All tests were two-sided, and p<0.05 was considered to indicate a statistically significant difference.

## Results

### General information

**Figure 1:**
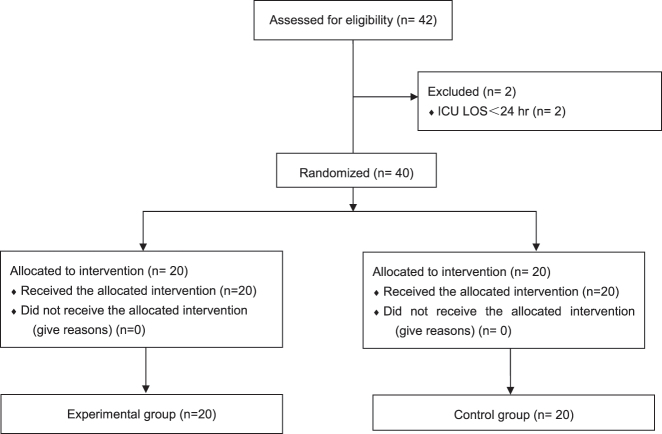
Flow diagram.

According to the inclusion and exclusion criteria, a total of 42 patients with sepsis and complicated acute kidney injury who required CRRT were included. Among them, 2 patients were excluded because their admission time was less than 24 h. Finally, 40 patients were included. All patients enrolled in the trial had a femoral venous catheter inserted on the right side. The computer random sequence list was randomly divided into an experimental group and a control group (the random sequence was placed in a sealed envelope with no light transmission), and the envelopes were numbered. When patients meeting the criteria were included, the envelopes were selected in order, and the patients were randomly grouped. General information is shown in Figure 1 and Table 1.

**Table 1:** Baseline information.

	Experimental group (n=20)	Control group (n=20)
Age, years	59.05 ± 17.28	60.07 ± 15.44
Sex, male	12	14
APACHE II score	13.05 ± 1.91	14.12 ± 0.73
MV	14	13
Anticoagulations/antiplatelets	3	2
Septic shock	12	10
Infection source		
Pneumonia	12	14
BSI	5	3
Other	3	3

APACHE II score, acute physiology and chronic health II score; BSI, bloodstream infection; MV, mechanical ventilation.

### Outcomes

At admission (experimental group vs. control group), the following parameters were found: sCr: 391.2 ± 1.94 vs. 389.7 ± 1.46 ummol/L, BUN: 12.97 ± 1.51 vs. 11.86 ± 1.75 mmol/L, APTT: 23.43 ± 1.27 vs. 24.12 ± 1.53 s, Plt: 80.15 ± 12.27 vs. 83.15 ± 10.35 × 109/L, 4 h after CRRT treatment: sCr: 280.85 ± 1.89 vs. 283.41 ± 1.46 ummol/L, BUN: 12.02 ± 1.55 vs. 11.86 ± 1.75 mmol/L, APTT of prefilter: 41 ± 3.89 vs. 25.31 ± 2.17 s, postfilter: 81.63 ± 3.76 s, Plt: 80.2 ± 13.04 vs. 83.15 ± 10.35 × 109/L, and pH value 7.37 ± 0.03 vs. 7.41 ± 0.02. See Table 2. A comparison of sCr and APTT (prefilter) revealed that p<0.05 was statistically significant, but there was no significant difference in the other indicators. In the control group, citrate accumulation occurred in 2 patients; for a total calcium/arterial calcium ion concentration >2.5, there were no obvious adverse reactions.

**Table 2:** Treatment variables (at admission).

Variables	Experimental group (n=20)	Control group (n=20)
sCr, umol/L	391.2 ± 1.94	389.7 ± 1.46
BUN, mmmol/L	12.97 ± 1.51	11.86 ± 1.75
APTT, s	23.43 ± 1.27	24.12 ± 1.53
INR	0.9 ± 0.2	0.8 ± 0.3
Hb	99 ± 8	97 ± 10
Plt, 109/L	80.15 ± 12.27	83.15 ± 10.35
pH	7.31 ± 0.04	7.33 ± 0.01
iCa, mmol/L	1.14	1.12
FF, %	18	17

Table 2-1 Treatment variables (4 h after CRRT)		
Variables	Experimental group (n=20)	Control group (n=20)
sCr, umol/L	280.85 ± 1.89	283.41 ± 1.46
BUN, mmmol/L	12.02 ± 1.55	11.86 ± 1.75
APTT, s		
Prefilter	41 ± 3.89	25.31 ± 2.17
Postfilter	81.63 ± 3.76	N/A
INR	0.8 ± 0.1	0.9 ± 0.3
Hb	96 ± 4	95 ± 4
Plt, 109/L	80.2 ± 13.04	83.15 ± 10.35
pH	7.37 ± 0.03	7.41 ± 0.02
iCa, mmol/L	1.12	1.13
Postfilter calcium ion, mmol/L	N/A	0.31

sCr, serum creatinine; BUN, blood urea nitrogen; APTT, activated partial thromboplastin time; Plt, platelet count; iCa, arterial calcium ion concentration; FF, filtration fraction; Hb, hemoglobin; INR, international normalized ratio.

### Outcomes

**Figure 2:**
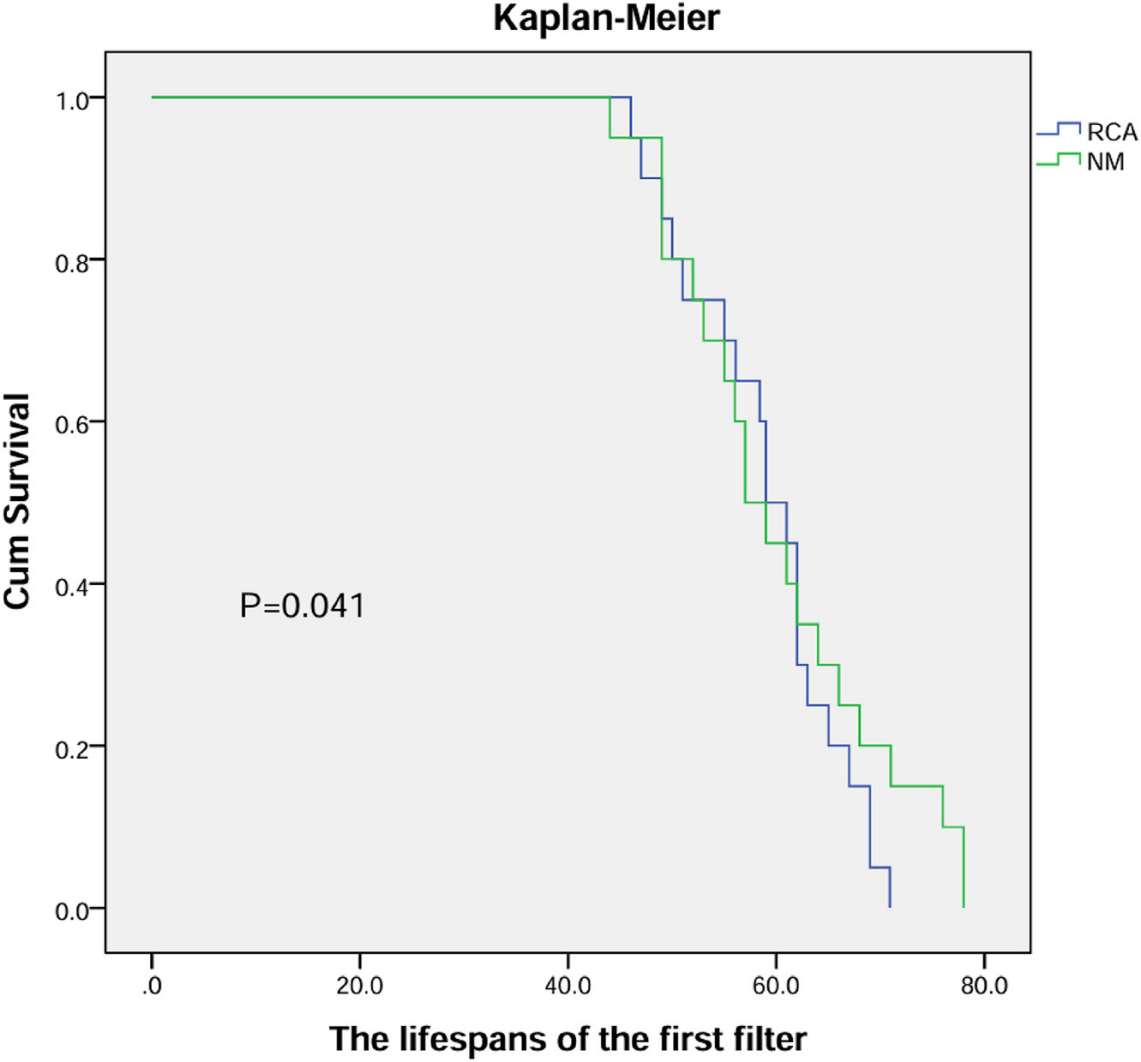
Lifespan of the first filter (h).

The lifespans of the first filter were 60.2 ± 10.09 and 59.07 ± 11.44 h, and the ICU lengths were 7.35 ± 1.35 and 7.21 ± 1.17 days. Nafamostat mesylate prolonged filter lifespan compared to RCA, however, ICU LOS was same. And no new bleeding events occurred in both groups. Details in Figure 2 and Table 3.

**Table 3:** Outcomes.

Variables	Experimental group (n=20)	Control group (n=20)
Lifespan of the first filter, h	60.2 ± 10.09^a^	59.07 ± 11.44
ICU length of day, days	7.35 ± 1.35	7.21 ± 1.17
Incidence of new bleeding episodes, n	0/20	0/20

^a^p<0.05.

## Discussion

According to the currently available data [12], 14], although the specific pathophysiological mechanism of sepsis-associated AKI is still unclear, it may be one of the earliest complications of sepsis. Like in sepsis research, over the past 20 years, experts worldwide have continued to explore and work hard to unify and standardize the definition of AKI. To date, the three largest classification systems have been developed: risk, injury, failure, loss, end-stage (RIFLE) criteria, acute kidney injury network (AKIN) criteria and the latest Kidney Disease: Improving Global Outcomes (KDIGO) standard [15]. The above three classification systems all rely on increased SCr levels and/or decreased urine output (UO) to determine the diagnosis of AKI. Despite this, the current sepsis guidelines still recommend using the Sequential Organ Failure Assessment (SOFA) score to define AKI. However, there are still limitations based on the definition of the SOFA score because SOFA score does not distinguish between acute kidney disease and chronic kidney disease, nor does it take into account demographic differences in baseline sCr levels. In the absence of a consensus definition, sepsis-associated AKI is defined based on the current clinical and pathophysiological understanding as a clinical syndrome characterized by a sudden deterioration in renal function in the presence of sepsis and not otherwise explained, manifested by sCr elevated levels, oliguria, or both [16], 17]. In addition to conventional bundled treatment programs for sepsis, the most effective treatment for sepsis-associated AKI currently is CRRT, which can quickly and effectively correct the uremia caused by renal dysfunction, thereby eliminating time for comprehensive treatment of sepsis [18]. During CRRT, an anticoagulation strategy is particularly important. An appropriate anticoagulation strategy can not only ensure the continuity of treatment but also ensure the effectiveness of treatment [19]. However, there is currently no ideal anticoagulation plan; although systemic heparin anticoagulation, regional citrate anticoagulation and heparin-protamine anticoagulation are routinely used in clinical practice, each regimen has certain limitations. A prospective randomized controlled study demonstrated that in patients at high risk of bleeding undergoing CRRT, the use of NM anticoagulation compared to unfractionated heparin was significantly associated with a lower risk of bleeding (NM: 3.3 % vs. UFH: 27 %; OR: 0.09; p=0.04) [11]. As previously mentioned, nafamostat mesilate inhibits most coagulation factors and platelets, and its half-life is significantly shorter than that of heparin. It can be rapidly degraded by hepatic carboxylesterase without affecting coagulation function. Due to its small molecular weight, NM can be cleared through dialysis/filtration, exerting anticoagulant effects only in the extracorporeal circulation circuit while rapidly inactivating *in vivo*. Therefore, when using nafamostat mesilate for anticoagulation during CRRT, the risk of bleeding may be lower than that of heparin, demonstrating advantages of convenient anticoagulation management and higher safety. Thus, in cases of heparin-induced type II thrombocytopenia or contraindications to heparin use, nafamostat mesilate can be considered an ideal anticoagulant. Therefore, this study intends to use a nafamostat mesylate anticoagulation regimen in patients with sepsis-associated AKI receiving CRRT to further clarify its safety and effectiveness. This study showed that the lifespan of the first filter of the included patients was 60.2 ± 10.09 h, which was basically equivalent to the 59.07 ± 11.44 h of regional citrate anticoagulation in the control group, basically indicating that the expected therapeutic effect was achieved. Second, 4 h after CRRT, no obvious abnormalities were found in coagulation indices, and no new bleeding events occurred in both groups. Its safety has been proven. Previous studies [20], 21] have shown that the NM anticoagulation strategy has significant advantages in patients at high risk of bleeding. However, our study found no bleeding events in either group, which may be related to the fact that the patients we included were not at high risk of bleeding. Patients with sepsis often suffer from multiple-organ failure. Therefore, the application of regional citrate anticoagulation is subject to certain limitations, such as hyperlactatemia, hypoxia, and liver insufficiency [22]. However, nafamostat mesylate, due to its ultrashort-acting effect, is rapidly metabolized. Therefore, this technique is suitable for use in patients with sepsis.

## Limitations

Nonetheless, our study has the following limitations. First, the sample in the present study was small, and it may not be possible to continue to administer this anticoagulation regimen to all sepsis-associated AKI patients. The sample size needs to be expanded in the future. To further clarify its safety and effectiveness in patients with sepsis-associated AKI. Second, the anticoagulation plan adopted in this study involved nafamostat mesylate at a bolus dose of 10 mg, and the initial dose was 20 mg/h. The APPT value of postfilter was maintained at 1.5–2 times the normal value. Although this approach is still classified as an empirical scheme, future trials should further establish different dose groups to further clarify the bolus dose and maintenance dose.

## Conclusions

Compared to the regional citrate anticoagulation strategy, the implementation of the NM anticoagulation strategy in patients with sepsis-induced acute kidney injury prolonged the filter lifespan without increasing the risk of bleeding. Therefore, the NM anticoagulation strategy is safe and effective. However, due to the small sample size, further expansion of the sample size is needed for validation.
